# Primary School Students with Reading Comprehension Difficulties and Students with Learning Disabilities: Exploring Their Goal Orientations, Classroom Goal Structures, and Self-Regulated Learning Strategies

**DOI:** 10.3390/bs13020078

**Published:** 2023-01-17

**Authors:** Christina Kampylafka, Fotini Polychroni, Alexandros-Stamatios Antoniou

**Affiliations:** 1 Department of Philosophy, Pedagogy and Psychology, School of Philosophy, National and Kapodistrian University of Athens, 15703 Athens, Greece; 2 Department of Education, School of Education, University of Nicosia, 1700 Nicosia, Cyprus; 3 Department of Psychology, School of Philosophy, National and Kapodistrian University of Athens, 15703 Athens, Greece; 4 Department of Primary Education, National and Kapodistrian University of Athens, 10680 Athens, Greece

**Keywords:** learning disabilities, reading comprehension difficulties, goal orientations, personal goals, classroom goal structures, strategies of self-regulated learning, motivation

## Abstract

The aim of the present study was to investigate goal orientations and classroom goal structures and their relationship with strategies of self-regulated learning (SRL) in students with and without learning disabilities (LD) and reading comprehension difficulties (RCD). The sample consisted of 537 students attending the two last grades of primary school, fifth and sixth grade (Mage = 11.28 years, SD = ±0.59). Of these, 58 students were diagnosed with LD, and 70 students, after individually administered assessments in reading accuracy and reading comprehension, were assigned to the RCD group. Self-reported questionnaires were administered, assessing students’ personal goal orientations, classroom goal structures, and strategies of SRL. The results showed that students with LD and students with RCD scored lower in mastery orientation and higher in performance avoidance compared to their peers without difficulties (ND). LD students reported lower scores of adaptive strategies than their peers. In addition, the results confirmed the adaptive character of mastery-approach goals and mastery goal structures and the negative effects of performance-avoidance goals and performance goal structures on the adaptive strategies of SRL. Performance-approach goals predicted adaptive behaviors for all students, confirming the argument of an adaptive type of motivation. The findings of the current study highlight the importance of goal orientations and classroom goal structures for students’ SRL. Implications of the findings for enhancing motivation for students with LD and students with RCD are discussed.

## 1. Introduction

Self-regulated learning (SRL) constitutes an essential skill for learning and psychosocial adjustment of students of all ages [1,2]. According to the literature [3,4,5], learners are self-regulated to the degree that they are cognitively, metacognitively, motivationally, and strategically active participants in their own learning. Improving students’ self-regulation is critical for the educational process, especially for at-risk students. Achievement goal theory [6,7], one of the most prominent theories in the field of motivation and school psychology [8], also extended in the classroom [9,10], provides the conceptual framework for this study. Different goal orientations are associated with different, more or less adaptive behaviors in the school context [11,12].

Especially for students with learning disabilities (LD), for whom motivation is of primary importance for learning involvement and achievement, research on achievement goals is relatively limited and the findings conflicting [13]. Moreover, taking into account the shallow orthographic system of the Greek language, Greek students with LD face prominent difficulties in reading fluency and comprehension rather than solely in reading accuracy. The identification of learning disabilities usually takes place after the third year of primary school and usually follows the discrepancy criterion, including administering an intelligence test and reading, spelling, and writing standardized, and informal tests. (see Method for assessment process in students with LD). Apart from students with LD, there are also students with reading comprehension difficulties (RCD) almost in every classroom. Because of the nature of reading comprehension, these deficits are often not as easily detected as reading difficulties, and there is no universally agreed identification criterion, so, consequently, students are not assessed and supported on time [14,15]. Children with RCD are usually identified as having low scores in reading comprehension tasks, with typical reading accuracy and intelligence scores. There is evidence that children who struggle with reading employ fewer and less effective learning and self-regulating strategies and report decreasing motivation [16]. Nevertheless, there is limited evidence on the motivational profiles and self-regulation of children who struggle with reading comprehension with no apparent reading difficulties and who remain undetected for a long time. Drawing from the achievement goal theoretical model, the present study explores personal and classroom goals and their link to the self-regulating strategies of students with RCD compared to students with LD and a group with no difficulties.

### 1.1. Goal Orientations, Classroom Goal Structures, and Self-Regulation

Achievement goals are conceptualized as the reason or the purpose for which someone is engaged in an achievement situation [17]. According to the theoretical framework in achievement tasks, students can pursue two distinct types of personal goals [18]: *mastery goals,* which correspond to the opportunity to learn, acquire knowledge, and develop competency [10,11] and *performance goals*, which correspond to competence demonstration and social/peer comparison [7,19]. According to the 2 × 2 framework [20], by adding the dimensions of approach (desire to engage in a task) and avoidance (effort of non-involvement in a task), four distinct goal orientations were formed. Students with mastery-approach goals are usually engaged in school tasks motivated by personal interest, aiming at the improvement of their own skills, while students with mastery-avoidance goals focus on avoiding task-based or intrapersonal incompetence [21]. Nevertheless, research findings for mastery-avoidance goals are relatively limited compared to other types of goals [22,23]. On the contrary, attaining superior competence or outperforming others is the basic aim for students with performance-approach goals [18,24,25,26]. Finally, performance-avoidance goals focus on avoiding failure and avoiding demonstrating lack of ability relative to others [27,28].

Given that personal attitudes and students’ motivation toward learning tasks are gradually shaped by the influence received from familiar surroundings, including the school classroom setting and the opposite [29,30], goal achievement theory has been extended from the individual-level construct to the characteristics of the educational setting, forming *classroom goal structures* [9,24]. Classroom goals reflect the perceptions of students about the messages received by the educational practices promoted during the learning process, such as the lesson’s organization, structure, and evaluation process [27,31]. Although the majority of goal orientation studies have widely adopted the trichotomy framework, research on classroom goal structures followed the initial dichotomy framework of mastery versus performance goals [32], including mastery goal structures, which focus on knowledge acquisition and individual progress, and performance goal structures, which focus on school performance and social comparison [33].

Empirical research data have shown that adopting a goal orientation and being in a classroom that promotes a specific goal is associated with either positive or negative outcomes, creating a divided model of adaptive and maladaptive goals [34,35]. Μastery-approach goals are positively linked to internal motivation and adaptive patterns of behavior [36,37,38], such as attention, effort, on-task persistence, deep processing of information [39,40], effective help-seeking [41,42], self-effectiveness, and use of cognitive and metacognitive strategies [43,44,45]. Strategies aiming at deep processing and a better understanding of the cognitive material fit a more adaptive pattern of knowledge acquisition than those aiming to surface processing, temporary memorization, and retrieval, which are considered to be more maladaptive [46,47].

On the other hand, findings regarding performance-approach goals are contradictory [48,49]. Τhere is evidence of an absence of a link between this orientation and deep processing strategies, particularly for primary school pupils [11,50], or that performance-approach goals are associated with undesirable outcomes, including anxiety, negative affect [51] and surface learning strategies [52]. However, data exist showing that in the case of demanding learning tasks [53], performance-approach goals have, at times, predicted more strongly than mastery-approach goals grades and academic achievement [54,55], self-regulation [56], engagement, effort [57], and persistence [33,58] among people high in need for achievement [35]. Based on these patterns, researchers accepted the benefits stemming from adopting this goal and promoted the revision of achievement goal theory through multiple goals [59]. Through multiple goals, students take advantage of the positive effects of both mastery and performance goals, as they are not mutually exclusive, and each of them could be beneficial and useful in a different way [35]. It is possible that controversies over the effects of performance-approach goals are due to differences in definition [60]. More specifically, as both elements are reported in the definition, the performance-approach goals’ emphasis mostly demonstrates competence and earning favorable judgments or outperforming peers [20,61]. This distinction is maybe crucial for these goals’ outcomes, as normative comparison seems to evoke more engagement, interest, and effort [62,63].

Finally, performance-avoidance goals have been described mainly as maladaptive, as they are positively linked to negative outcomes in both cognitive and emotional domains [30,64], including self-handicapping strategies [65,66], instances of acquired helplessness, withdrawal [33,67], procrastination behavior [68], high anxiety [69], strategies of surface processing [70,71], and low achievement [28]. In addition, performance-avoidance goals were found to be negatively correlated with effective strategies of self-regulation [72] and school performance [73].

As with personal achievement goals, students’ perceptions of mastery classroom goals or goal structures have been associated with positive behavior patterns, such as mastery goals, deep strategies, effort, internal motives, help-seeking, support autonomy, and positive affect [74,75,76]. Mastery goal structures were found to be a positive predictor of deep-level learning strategies, critical thinking, metacognitive skills, effort, and school engagement [77,78,79]. In contrast, performance-oriented classrooms are linked to maladaptive educational behaviors, such as self-handicapping strategies, surface strategies, anxiety, and shame [67,71,80]. They are also negatively associated with internal motives, deep strategies, effective management of demanding tasks [24,30,81] and persistence [76].

### 1.2. Personal and Classroom Goals and Self-Regulated Learning of Children with LD and RCD

At-risk students or low achievers often present difficult motivational and behavioral profiles [82,83,84]. Nevertheless, relatively few studies have been conducted on personal goals, classroom goal structures, and their outcomes for students with LD or RCD [13,85,86,87,88,89]. Particularly, students with LD may report low levels of motivation and engagement in learning tasks [90], task avoidance [91], high levels of learned helplessness, academic procrastination, negative affect, self-handicapping, low levels of academic self-efficacy, and low help-seeking [13,92,93,94,95]. Empirical data also show that students with LD adopt high levels of performance-avoidance goals, low mastery-approach goals [13,96], perceive their class as more performance-oriented [85], use reduced self-regulating strategies, adopt surface approach to learning, present diminished persistence toward the learning goal, high levels of shallow cognitive processing strategies, low use of deep strategies, deficits in metacognitive skills, low levels of monitoring, and high anxiety [97,98,99,100]. Moreover, for students with LD, mastery-approach goals have been positively linked with adaptive motivational and behavioral outcomes, whereas performance-avoidance goals are associated with mostly maladaptive patterns [101], indicating similar outcomes with students without difficulties. For example, Sideridis [102] found that mastery-approach goals negatively predicted helplessness and positively predicted performance, while performance-avoidance goals positively predicted helplessness.

Struggling readers present a similar motivational profile to LD students reporting low motivation, negative attitudes toward reading, surface approach to learning, poor self-regulation skills, and low use of deep approach as compared to skilled readers [103,104,105]. Especially for poor comprehenders, empirical findings indicate deficits in motivation and working memory, low levels of monitoring, lower use of evaluation/integration and self-regulation strategies, lower school enjoyment, and higher levels of burnout than typical students [106,107,108,109]. Moreover, students with reading disabilities are significantly more performance-avoidant compared to typical students [110].

Although the effects of performance-approach goals are not sufficiently clear [111], it is argued that performance-approach goals are more adaptive for high-risk students, since they are positively associated with effort and school performance [102,112]. In addition, performance-approach goals are more adaptive for students with low self-perceived competence [35,113]. Finally, there are a few results highlighting the importance of mastery goal structures in reading comprehension [32], indicating that performance goal structures are associated with less positive affect and less engagement, adopting the opposite pattern of mastery goal structures [114].

### 1.3. The Present Study

In the present study, we examined goal orientations, classroom goal structures, and strategies of SRL in three separate groups: students with LD, students with RCD, and students with no difficulties, with the aim of describing their motivational profiles. A second aim was to explore the different patterns of predictors for adaptive and surface strategies for the three groups, aiming to investigate the outcomes of personal goals and classroom goal structures. In this context, we explored the adaptive or non-adaptive role of performance-approach goals on self-regulation. The literature regarding the effects of this personal goal has been inconclusive, on the one hand supporting the adaptive character of the goal and, on the other, promoting the negative outcomes [56,115].

In this study, the emphasis is given to students with LD and students with RCD, given the few and inconsistent findings of previous studies that have not led to a clear pattern for students facing difficulties [49,116]. Most of the existing literature for students with RCD focuses on the fundamental skills of the reading process [117,118,119], disregarding the psychosocial ramifications of this specific difficulty [84]. Furthermore, motivation and SRL in relation to reading comprehension skills have been rather neglected in empirical studies [120,121]. Finally, the sample was primary school students since they have received relatively limited research attention regarding these variables [122] compared to high school and University students [123,124,125].

Taking into account the existing literature, the research hypotheses and research question were formed as below:

1. It is more likely that students with LD and students with RCD would report lower scores of mastery-approach goals and mastery goal structures and higher performance-avoidance goals and performance goal structures than students without difficulties;
2. It is more likely that students with LD and students with RCD would report lower levels of adaptive self-regulating strategies (i.e., deep, motivational, persistence, and monitoring) and higher levels of surface strategies as compared to students with no difficulties;
3. In terms of differences between the LD group and the RCD group, it is less likely that LD students would present an adaptive profile, reporting fewer mastery goals, more performance-avoidance goals, more performance goal structures, and fewer adaptive strategies;
4. Are personal goals and classroom goal structures predictors of SRL strategies, and which of them is a predictor of adaptive and surface strategies separately for the three groups?

## 2. Materials and Methods

### 2.1. Participants and Procedure

Five hundred and thirty-seven students attending the 5th and 6th grades of 28 public primary schools in Athens, Greece, participated in the study, selected from a larger pool of 568 students. The mean age of the participating students was 11.28 years (SD = ±0.59, Min = 10 years, Max = 12.75 years). All students were native speakers of Greek. Of them, 58 students had a formal statement of learning disabilities (LD group; 31 fifth graders and 27 sixth graders; 41 boys and 17 girls) by the state assessment Centers for Differential Diagnosis, Diagnosis, and Support (KEDASY). To get a formal statement of learning disabilities, the following criteria were met: average intelligence using the WISC-III test [126] (standardized in Greek), low reading ability (decoding and fluency) as measured by standardized and informal tests and no other coexisting neurodevelopmental difficulties. The LD group included children with average and above-average comprehension abilities. For the reading comprehension group (RCD group), 70 students from the total sample, 42 fifth graders and 28 sixth graders, 36 boys and 34 girls (excluding the students identified with LD), were selected after individualized assessments. For inclusion in the RCD group, the following criteria were met: students (a) scored at or above the 25th percentile on reading accuracy, (b) scored lower than the 25th percentile on reading comprehension [119,127] (see the measures in the next section) following the criteria used in other relevant empirical studies [128,129], and (c) had no known coexisting difficulties. Students included in the RCD group were not included in the LD group, and vice versa. The third group of the study consisted of 409 students (189 fifth graders and 220 sixth graders; 191 boys and 218 girls) with no formal statement and no other known difficulties according to their teachers and comprised the non-difficulties (ND) group. Finally, it should be stated that students who according to their classroom teachers’ perceptions had reading difficulties or were in the process of assessment for any other developmental disorder were not included nor in the ND group or in the RCD group.

### 2.2. Measures

#### 2.2.1. Personal Goal Orientations

The Questionnaire of Achievement Goal Orientations [96,102] was used to assess students’ personal achievement goals. The questionnaire is domain specific (language) and consists of 18 items with three subscales, i.e., Mastery-Approach Goals-MAP goals- (6 items; e.g., “How important is to you to understand the language course?”), Performance-Approach Goals-PAP goals- (6 items; e.g., “How important is to you to get the best grade in the language course?”), and Performance-Avoidance Goals-PAV goals- (6 items; e.g., “Are you worried that you might not get a high grade in the language course?”). Τhe mastery-avoidance subscale (3 items) was not included in the present study since it is argued that this construct is more present in academic and competitive settings and less so in young students and different settings, especially in elementary school students [22,130,131]. A four-point Likert-type scale (from 1 = not at all to 4 = very much) was used. Cronbach’s alpha for MAP goals was α = 0.88, α = 0.92, and α = 0.91; for PAP goals α = 0.86, α = 0.92, and α = 0.83; and, finally, for PAV goals was α = 0.74, α = 0.90, and α = 0.74 (for ND, LD, and RCD groups, respectively).

#### 2.2.2. Classroom Goal Structures

Classroom goal structures were assessed with the Questionnaire of Classroom Goal Structures [114]. Its items derived from a synthesis of scales (e.g., the Patterns of Adaptive Learning Scales-PALS) [31,67]. The questionnaire includes 16 items, and the students select a response from a four-point Likert-type scale, ranging from 1 (Not at all) to 4 (Very much). The questionnaire contains 2 subscales, Mastery Goal Structures (M-STR) (e.g., “The teacher tells us that mistakes don’t matter”) and Performance Goal Structures (P-STR) (e.g., “During the lesson, there is a lot of competition between students”) (M-STR α = 0.89, α = 0.85, α = 0.91; P-STR α = 0.87, α = 0.91, α = 0.89 for ND, LD, RCD groups, respectively).

#### 2.2.3. Strategies of Self-Regulated Learning

Children’s Perceived Use of Self-Regulated Learning Inventory [132] is a self-report tool that assesses students’ self-regulation in school tasks. The questionnaire includes 75 items in a 5-point Likert-type scale from 1 (Never) to 5 (Always) that examine 9 basic components of SRL, i.e., task orientation, planning, motivation, self-efficacy, monitoring, learning strategies, motivational strategies, persistence, and self-evaluation. For the purpose of the present study, four subscales were used, learning strategies, motivational strategies, persistence, and monitoring. The learning strategies contain 14 items grouped into two factors, the *surface strategy* with four items (e.g., “When studying, I read or recall everything again and again until I know it by heart”) (α = 0.71, α = 0.74, α = 0.75 for ND, LD group, and RCD, respectively) and the *deep strategies* with 10 items (e.g., “When studying, I make a scheme or a mind map”) (α = 0.87, α = 0.90, α = 0.91 for the three groups, respectively). *Motivational strategies* contain 4 items (e.g., “During my schoolwork, I say to myself: You can do it, just keep on working!”) (α = 0.82, α = 0.79, and α = 0.87 for ND, LD, and RCD group, respectively), *monitoring* contains 7 items, (e.g., “If I notice something isn’t working out, I try a different approach”) (α = 0.83, α = 0.79, and α = 0.89 for ND, LD, and RCD group, respectively), and *persistence* includes 6 items such as “Even if I would rather do other things, I finish my schoolwork (α = 0.80 for ND group, α = 0.91 for LD group, and α = 0.92 for RCD group). In accordance with the theoretical models, deep strategies, motivational strategies, monitoring, and persistence were categorized as adaptive self-regulating strategies.

#### 2.2.4. Reading Accuracy

A word reading task and a non-word reading task of the Greek standardized Reading Test TEST-A [133] were administered individually as indicators of reading accuracy. The word reading task consists of 53 isolated words, and the non-word reading task consists of 24 non-words printed in two columns. Both words and non-words are presented in order of difficulty. The test is discontinued when children score zero on five consecutive items (α = 0.86, α = 0.74, and α = 0.76 for ND, LD, and RCD group, respectively).

#### 2.2.5. Reading Comprehension

Two short informative texts, from the reading comprehension task of the Reading Test TEST-A [133] were administered to the students. Students were asked to read the texts aloud or silently and then to respond to six multiple choice literal, vocabulary-dependent, and inferential questions. The number of correct responses in both texts was employed as measure of comprehension (α = 0.72, α = 0.78, and α = 0.73 for ND, LD, and RCD group, respectively).

### 2.3. Procedure and Ethics

The questionnaires and individual assessments were administered to students during school hours by the researcher. Students completed the self-report questionnaires in the classroom, and then they were assessed individually in reading accuracy and comprehension tasks. Students were assisted with their reading by the researcher, if necessary, especially in the case of students with learning disabilities. The study was approved by the Hellenic Institute of Educational Policy of the Ministry of Education, a body that granted consent for access to schools, and parental consent was a prerequisite for students’ participation in the study. Students having parental consent were also asked if they were willing to participate in the study, and they were informed that they had the opportunity to withdraw from the study at any stage.

## 3. Results

### 3.1. Descriptives and Group Comparisons

Kruskal–Wallis tests were performed to test for differences in median scores on goal orientations and classroom goal structures between the three groups of students (ND, LD, RCD) as the normality hypothesis was rejected for all variables (Shapiro–Wilk normality test). There were statistically significant differences between the medians of the groups for all the goal orientations and classroom goal structures (*p* < 0.05). 

Post hoc analyses based on Mann–Whitney U test pairwise comparisons showed that students with LD and students with RCD reported significantly lower MAP goals, M-STR, and higher PAV goals and P-STR as compared with the ND group. Moreover, LD students reported significantly lower PAP goals as compared with the ND group. Finally, LD students presented lower MAP goals, higher PAV goals, and P-STR as compared to RCD group (Table 1).

### 3.2. Predictors of Surface and Adaptive Strategies

Pearson’s r coefficients for the study variables are presented in Table 2 for the three groups of the study. The correlations between the predictor variables of the study were examined, and they were found to be low to moderate, indicating that collinearity was unlikely to be a problem [134].

The results for all groups showed that MAP goals and M-STR were positively correlated to adaptive strategies and negative correlated to surface strategies of SRL. PAP goals were also positively linked to adaptive strategies. Finally, PAV goals and P-STR were negatively linked to adaptive strategies and positively linked to surface strategies.

For each one of the two dependent variables (Surface and Adaptive Strategies), a series of linear regressions analyses was run to estimate the effect of each one of the predictors (MAP, PAP, PAV, M-STR, P-STR, Group, Gender, Class) and their possible interactions on the dependent variables. The assumptions of the linear regression analysis were not violated (normality assumption, assumption of equal variance, multicollinearity as GVIF values <10). Starting from “Surface Strategies”, a linear regression model that included all the main effects was conducted. Moving forward, we eliminated all the predictors that did not have a statistically significant effect-each time, the predictor with the highest non-significant p-value was excluded-and then tried to add only significant interaction terms.

The procedure was repeated until the Model 5 (*F*(5, 531) = 140.579, *p* = 0.000, R^2^ = 0.570) included only statistically significant main effects (Table 3). To evaluate if the latest reduced model (Model 5) was similar to the initial full one (Model 1), an Anova test was utilized. No statistically significant difference was found in the fit between Model 1 and Model 5 since *p* = 0.834 > α = 0.05, so Model 5 consisted the base to identify possibly significant interactions (MAP and Group, PAP and Group, P-STR and Group).

Table 4 shows that only in Model 8 (*F*(7, 529) = 102.809, *p* = 0.000 R^2^ = 0.576) the group RCD by P-STR term had a statistically significant effect on surface strategies (*p* = 0.005 < α = 0.05). It was observed that, for the models 6 and 7 that contained the other two possible interaction terms, the interaction terms did not have a statistically significant effect on surface strategies (both *p* > 0.05).

The final model was Model 8, as the ANOVA tests comparison showed that Model 8 had a statistically significantly better fit than Model 5 (*p* = 0.017 < α = 0.05). Looking into Model 8, it was observed that there was a positive effect of MAP, PAP, and P-STR on surface strategies, and more specifically, if MAP was increased by one unit, then the surface strategies was increased by 0.097 on average. Similarly, if PAP was increased by one unit, the surface strategies was increased by 0.490 ceteris paribus. In the case of the ND group (level of reference of group), when P-STR was increased by one unit, the surface strategies was increased by 0.363 on average, ceteris paribus. Furthermore, we observed that also for the LD group, P-STR had a similar effect on surface strategies with the ND group since the interaction term group LD by P-STR was not statistically significant (*p* = 0.854 > α = 0.05). On the other hand, for the RCD group, the interaction term group RCD by P-STR was statistically significant (*p* = 0.005 < α = 0.05) with a positive coefficient of 0.257, which means that the increase rate in the surface strategies was higher than the other two groups (0.36 + 0.26 = 0.62) when the P-STR was increased by one unit. It should be noted that on average, the group RCD had a lower coefficient (−0.411) as an initial value compared to ND (level of reference) and LD (0.518). Bringing together the main group effect and the interaction term, regarding the RCD group, for low values of P-STR, the surface strategies value was lower than the other two groups but as P-STR increased, the surface strategies increase was more aggressive than for the other two groups. To better visualize the interaction effect of P-STR and groups on surface strategies, we utilized the sjPlot R package [135] to construct the plot of surface strategies with respect to P-STR for each group separately.

**Figure 1:**
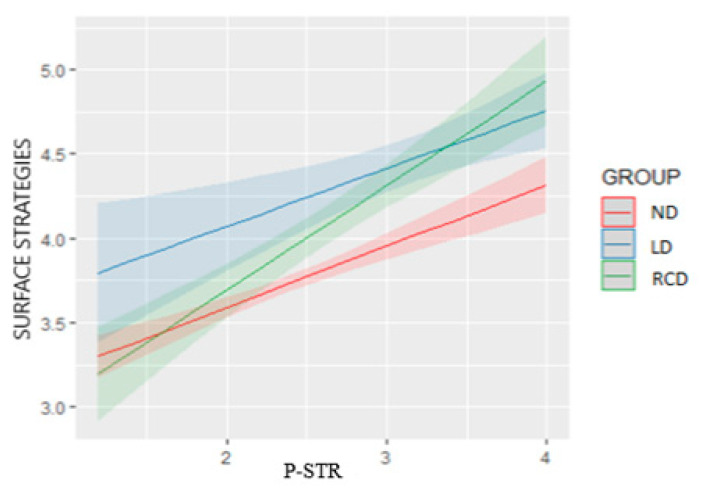
Interaction effect of P-STR and Groups on Surface Strategies.

As expected, the RCD green line had lower surface strategies values than the other two groups for low P-STR values, but it had a higher slope, and it crossed the two other lines, resulting in higher values of surface strategies than the two other groups for high values of P-STR (Figure 1).

Passing now to adaptive strategies, a linear regression model that included all the main effects of the adaptive strategies was conducted. As in the case of surface strategies, we excluded all the predictors that did not have a statistically significant effect and then added only significant interaction terms. 

As Model 2 (Table 5) consisted of only the statistically significant terms (*F*(8, 528) = 199.570, *p* = 0.000, R^2^ = 0.751), an ANOVA test was utilized to evaluate if the latest model (Model 2) showed a similar fit to the initial one (Model 1). No statistically significant difference was found in the fit between Model 1 and Model 2 (*p* = 0.8054 > 0.05), so Model 2 was used as a base to identify significant interactions (PAP and Group, MAP and Group, PAV and Group, M-STR and Group, P-STR and Group).

Running linear regressions models with interactions, we observed that only the Group LD by MAP interaction term in Model 4 (Table 6) (*F*(10, 526) = 162.208, *p* = 0.000, R^2^ = 0.755) had a statistically significant effect on adaptive strategies (*p* = 0.011 < α = 0.05). All the other models did not have any extra significant terms.

An Anova test was utilized to examine if the fit of Model 4 is statistically significantly better than Model 2, which contains all the main effects. Since *p* = 0.020 < α = 0.05, we concluded that Model 4 with the interaction term of Group LD by MAP had a statistically significantly better fit to the data.

**Figure 2:**
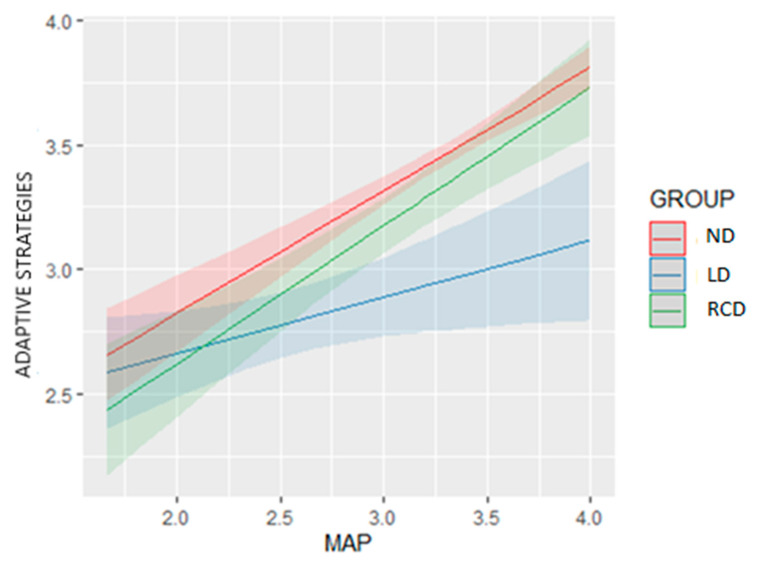
Interaction effect of MAP goals and Groups on Adaptive Strategies.

As far as Model 4 is concerned, there was a main positive effect of MAP on adaptive strategies for the level of reference ND of Group, where the adaptive strategies score was increased by 0.493 when the MAP was increased by one unit. For the RCD group, a similar pattern was observed as the interaction term was small and non-significant (*p* = 0.477 > α = 0.05). However, for the LD group, there was a negative interaction term MAP by group (−0.266) that decreased the overall positive effect of MAP on the adaptive strategies score (0.493 − 0.266 = 0.227). So, for the LD group, a unit increase in MAP brought a 0.227 increase in adaptive strategies. The above differences in the slopes of MAP for each group level are also presented in the estimated marginal means interaction plot below, where the blue line (LD group) has a smaller slope than the slopes of the other two groups (Figure 2).

### 3.3. Descriptives and Group Comparisons Based on Final Models

For an overall comparison of the mean Surface and Adaptive Strategies between the three groups (ND, LD, RCD), the estimated marginal means together with their 95% confidence intervals were calculated (Table 7) and pairwise Tukey comparisons between the three groups were conducted using the “emmeans” R package [136].

From the Tukey tests, we concluded that the mean ND surface strategies were statistically significantly lower than the ones of LD and RCD (*p* < 0.0001 and *p* = 0.0021 < 0.05), and marginally, there is no statistically significant difference between LD and RCD surface strategies (*p* = 0.0641 < α = 0.05). Moreover, based on the estimated marginal means comparison t-test with Tukey adjustment, it was observed that the LD group had a statistically significantly lower adaptive strategies score than the two other groups (*p* < 0.001 and *p* = 0.003), while the ND and RCD groups showed similar performance (*p* = 0.124).

## 4. Discussion

The aim of the present study was to explore self-reported goal orientations, classroom goal structures, and strategies of self-regulated learning of students with learning disabilities, students with reading comprehension difficulties, and students with no difficulties. Moreover, we examined the predictors of adaptive and surface strategies for the three groups.

The results of the present study showed that the students with learning disabilities and the students with comprehension difficulties were less mastery and more performance-avoidant-oriented and reported higher levels of performance goal structures as compared to students without difficulties. This result confirms the first hypothesis and is in line with extant research showing that students with learning disabilities and poor comprehenders present low mastery goals and avoid learning tasks because of their fear of poor performance and deficits exposure, and they usually perceive the classroom context as performance-oriented [85,137].

In terms of self-regulated strategies, students with learning disabilities and reading comprehension difficulties reported more surface strategies than their typical peers, partly confirming the second hypothesis and the existing literature [90,138]. Moreover, only the group with learning disabilities reported lower scores on the adaptive self-regulating strategies (deep, motivational, monitoring, and persistence) as compared to students with no difficulties. This finding partly confirms the second hypothesis and agrees with previous studies showing that students with learning disabilities report lower levels of self-regulation strategies than typical peers [97,139]. Nevertheless, poor comprehenders showed similar use of adaptive strategies to students without difficulties, contrary to the bulk of evidence showing the use of fewer self-regulating strategies [106]. This may imply that these students may try to use adaptive strategies, but since these are particularly demanding, they may not be effective and lead to low comprehension performance. These findings underline the motivational deficits of students with learning disabilities and comprehension difficulties, highlight the importance of enhancing their self-regulation for learning and turn the focus on students with comprehension difficulties, a group whose difficulties remain undetected and are largely underestimated [14]. In the case of students with comprehension difficulties, suitable decoding skills might mask comprehension deficits, and low performance in comprehension tasks is often attributed to external causes, such as low attention or lack of effort [128]. For poor comprehenders, being intrinsically motivated and using effective strategies of self-regulated learning during the reading process can lead to the successful completion of a comprehension task. Therefore, further research is needed using methods such as behavioral observation tasks for students with comprehension difficulties, as these groups frequently do not have a formal special educational needs statement and whose self-regulation profile is not straightforward.

Regarding the differences between the students with learning disabilities and the students with comprehension difficulties, the former group reported lower levels of mastery-approach goals, higher levels of performance-avoidance goals and performance goal structures, and fewer adaptive strategies than their peers with poor comprehension. These findings confirm our third hypothesis and are in agreement with other studies showing that students with learning disabilities are a highly heterogeneous group, facing a wide range of deficits in motivational, behavioral, cognitive, and psychosocial characteristics [140,141]. As far as their goal orientation is concerned, given that they have difficulties in more than one learning area [90,142], it is expected that they adopt fewer mastery-approach goals and more performance-avoidance goals, probably in order to avoid exposure of their weak areas [143]. On the other hand, students with comprehension difficulties present difficulties mainly in one specific domain [118].

The present study also explored the different patterns of predictors for strategies of self-regulated learning for the three groups, looking into the prediction of more and fewer adaptive strategies. Regarding the research question, the results highlighted the importance of motivation (personal and contextual) by adopting a personal goal or being in an environment that promotes specific goals for self-regulated learning of all students, regardless of the type of difficulty [144]. Mastery-approach goals and mastery classroom goals were positive predictors of adaptive strategies of self-regulated learning for all three groups (typical students, students with learning disabilities, and students with reading comprehension difficulties). This finding confirmed that mastery orientation personal goals and classroom mastery orientation goals are associated with positive behavior models at a cognitive level, not only for the group without any known difficulties but also for students with learning disabilities and reading comprehension difficulties. In essence, students who estimate that the educational practices in their class are focused on learning and who, on their own, aim at self-improvement might be led to the use of more strategies that aim at in-depth processing of the learning material and demonstrate higher levels of motivational strategies, monitoring, and persistence. This result concurs with other findings and confirms the adaptive character of mastery goals and the corresponding classroom goal structures for all students [8]. Given that mastery goal structures are associated with adaptive self-regulatory strategies, such as monitoring, deep, and motivational strategies, these should be reinforced at school [55,114]. Reinforcing mastery goal structures will probably increase the adoption of personal mastery goals since the school setting, and the classroom’s practices seem to affect the students’ personal attitudes and behaviors [10]. In the case of LD students, mastery goals had a lower impact as a predictor of adaptive strategies. This finding is probably due to the fact that students with learning disabilities are less oriented to learning, adopt lower mastery goals than other students, and their mastery goals are not stable during learning tasks. As a result, they have very low engagement in a learning task and avoid using deep and complex strategies for it [90].

However, the reverse pattern was observed in performance-avoidance goals. Avoiding learning engagement for fear of low performance and adopting performance-avoidance goals were found to be positively linked to surface strategies and were negatively linked to adaptive strategies. More specifically, performance-avoidance goals negatively predicted adaptive strategies of self-regulated learning in all groups. This result confirms the extant literature that supports the less adaptive character of one’s performance-avoidance goals toward learning behaviors and school performance [28,70,96]. Performance goal structures positively predicted surface strategies, and they were also negatively linked to adaptive strategies of self-regulating learning for all groups. This finding concurs with previous research [24,71,76] for typical students and both at-risk groups. On the other hand, for students with comprehension difficulties, performance classroom goals were a stronger predictor of surface strategies. Taking into account that poor comprehenders’ difficulties are detected in a specific domain, these students may better adapt and respond according to the classroom’s orientation. In other words, in a performance-oriented classroom, they might use more surface strategies since they are simpler and easier strategies. However, further research is also warranted. On the whole, the results regarding performance classroom goals seem to justify the researchers who have considerable reservations about the introduction of such structures into the educational setting [81].

According to the present study, performance-approach goals significantly predicted surface strategies for all groups, in agreement with the literature [51]. Moreover, performance-approach goals, whose role is questionable [61], were a positive predictor of adaptive strategies for all groups. This finding is consistent with studies showing that performance-approach goals could be effective for all students, leading to the adoption of more adaptive behavioral models [61,115]. Additionally, researchers claim that students with learning disabilities and students with reading comprehension difficulties or low-performance students, by adopting performance-approach goals, are motivated toward the achievement of a specific goal and refrain from task resignation or avoidance, so even this presence of motivation becomes beneficial as compared to the absence of any motives [88]. Moreover, awareness of shortcomings and elevated stress levels due to fear of exposure to weaknesses and desire for higher performance, apart from negatively affecting psychosocial adjustment and well-being [51], could possibly intensify effort and lead to the use of more effective strategies [145]. It might facilitate our understanding of the results if we take into consideration that the performance-approach goals can include both normative and appearance standards and that the normative standards activate the use of self-regulating strategies [146]. Consequently, this finding highlights that avoidance orientation is the one negatively linked to adaptive behaviors for all students, while the effect of performance-approach goals warrants further research.

Taking the above results into consideration, emphasis is put on the value of classroom goals and personal mastery goals for learning, but also on the negative effect of personal performance-avoidance goals for all students, regardless of their presented difficulties. Promoting mastery of classroom goals and therefore encouraging students to adopt personal goals of acquiring knowledge and improving themselves by individual standards is of the essence in the classroom [80]. At the same time, the importance of performance-approach goals for more adaptive learning behaviors, in the case of poor-performance students, is not to be understated. The above findings are in favor of promoting educational orientation toward learning in the classroom, while further investigation is required concerning the role played by performance-approach goals to the performance of students with learning disabilities and comprehension difficulties. Furthermore, these results are in agreement with studies that favor the revision of the goal orientation theory since they demonstrate that performance-approach goals do not necessarily lead to negative behavior models [59]. Consequently, multiple goals, which aim both at learning and performance, are more likely to lead to a more adaptive behavioral model [26,147].

### Limitations and Conclusion

The use of self-report may lead students to report strategies that they have not used, offering socially desirable responses, or that they might be unaware of strategies they used automatically. It is equally probable that the individual characteristics of students are incorporated into the perceived classroom goals, thus highlighting the subjective nature of the interpretation of educational practices within the classroom. For example, the relationship between teachers and students or between peers could be an important factor affecting students’ attitudes toward the school environment; however, this was not taken into account in the present study [122]. Future studies may employ rating scales by teachers and parents and behavioral observation as additional methodological tools. In addition, the learning disabilities group included students with a formal statement of learning disabilities, without further assessment by the authors. Students might present difficulties in other learning areas, and the majority of these might have already participated in intervention programs, and this may have an effect on the reported strategies. It should also be noted that students identified with poor reading comprehension were only selected by the pool of students with no known difficulties following reading comprehension tasks. This therefore, did not explore students’ other possible learning needs.

These findings of the present study expand our knowledge of achievement goals and self-regulated learning strategies in students with comprehension difficulties and students with learning disabilities. Given the importance of motivation in the learning process, the results of the present study may have implications for the identification of the motivational profile of at-risk students and the implementation of primary and secondary prevention programs with the aim of forming self-regulated learners.

## Figures and Tables

**Table 1:** Mean scores, median scores, and standard deviations for the study variables.

Personal Goals and Classroom Goal Structures	ND (*n* = 409)			LD (*n* = 58)			RCD (*n* = 70)			χ^2^ (*df* = 2)
	M	Mdn	S.D.	M	Μdn	S.D	M	Mdn	S.D.	
MAP	3.44	3.67 _a_	0.55	2.42	2.25 _b_	0.60	2.94	2.83 _c_	0.68	111.64 **
PAP	2.94	3.00 _a_	0.67	2.67	2.58 _b_	0.71	2.78	2.66 _ab_	0.60	10.76 *
PAV	2.72	2.67 _a_	0.60	3.55	3.83 _b_	0.56	3.19	3.17 _c_	0.60	89.27 **
M-STR	3.22	3.43 _a_	0.65	2.54	2.50 _b_	0.52	2.58	2.43 _b_	0.78	80.48 **
P-STR	2.32	2.11 _a_	0.64	3.19	3.11 _b_	0.63	2.68	2.72 _c_	0.67	76.59 **

Notes: Median scores that share the same index (a,b,c) are not statistically different according to the post hoc test Mann–Whitney *U* test for α = 0.05. MAP = mastery-approach goals; PAP = performance-approach goals; PAV = performance-avoidance goals; M-STR = mastery goal structures; P-STR = performance goal structures. * *p* < 0.05 ** *p* < 0.001.

**Table 2:** Correlations of personal goals, classroom goal structures, and strategies of self-regulated learning.

Personal Goals and Classroom Goal Structures	Strategies of SRL	
	Surface	Adaptive
MAP	−0.23 **	0.80 **
PAP	0.62 **	0.07
PAV	0.47 **	−0.64 **
M-STR	−0.25 **	0.76 **
P-STR	0.64 **	−0.53 **

Note: MAP = mastery-approach goals; PAP = performance-approach goals; PAV = performance-avoidance goals; M-STR = mastery goal structures; P-STR = performance goal structures. ** *p* < 0.001.

**Table 3:** Model 5: Linear regression analysis for predicting surface strategies.

Predictors	Surface Strategies			
	B	S.E.	*t*	*p*
(Intercept)	1.07 **	0.20	5.47	0.000
MAP	0.10 *	0.04	2.19	0.029
PAP	0.49 **	0.04	12.19	0.000
Group LD	0.42 **	0.08	5.39	0.000
Group RCD	0.26 **	0.06	4.10	0.000
P-STR	0.40 **	0.05	8.47	0.000

Note: MAP = mastery-approach goals; PAP = performance-approach goals; P-STR = performance goal structures. * *p* < 0.05 ** *p* < 0.001.

**Table 4:** Model 8: Linear regression analysis for predicting surface strategies.

Predictors	Surface Strategies			
	B	S.E.	*t*	*p*
(Intercept)	1.13 **	0.20	5.72	0.000
MAP	0.10 *	0.04	2.26	0.024
PAP	0.49 **	0.04	12.36	0.000
Group LD	0.52	0.33	1.57	0.116
Group RCD	−0.41	0.25	−1.68	0.094
P-STR	0.36 **	0.05	7.21	0.000
P-STR: Group LD	−0.02	0.10	−0.18	0.085
P-STR: Group RCD	0.26 *	0.09	2.83	0.005

Note: MAP = mastery-approach goals; PAP = performance-approach goals; P-STR = performance goal structures. * *p* < 0.05 ** *p* < 0.001.

**Table 5:** Model 2: Linear regression analysis for predicting adaptive strategies.

Predictors	Adaptive Strategies			
	B	S.E.	*t*	*p*
(Intercept)	0.84 **	0.22	3.79	0.000
MAP	0.47 **	0.05	9.10	0.000
PAP	0.16 **	0.03	4.84	0.000
Group LD	−0.30 **	0.07	−4.16	0.000
Group RCD	−0.16 *	0.06	−2.55	0.011
PAV	−0.24 **	0.04	−5.47	0.000
Μ-STR	0.40 **	0.04	9.08	0.000
Gender Girl	0.13 **	0.04	3.36	0.001
Class 6th	0.08 *	0.04	2.20	0.028

Note: MAP= mastery-approach goals; PAP= performance-approach goals; PAV= performance-avoidance goals; M-STR= mastery goal structures. * *p* < 0.05 ** *p* ≤ 0.001.

**Table 6:** Model 4: Linear regression analysis for predicting adaptive strategies.

Predictors	Adaptive Strategies			
	B	S.E.	*t*	*p*
(Intercept)	0.83 **	0.23	3.61	0.000
MAP	0.49 **	0.06	8.90	0.000
Group LD	0.37	0.28	1.31	0.190
Group RCD	−0.32	0.27	−1.21	0.228
PAP	0.16 **	0.03	4.94	0.000
PAV	−0.25 **	0.04	−5.75	0.000
Μ-STR	0.38 **	0.04	8.74	0.000
Gender Girl	0.13 **	0.04	3.25	0.001
Class 6th	0.09 *	0.04	2.25	0.025
MAP: Group LD	−0.27 *	0.11	−2.54	0.011
MAP: Group RCD	0.06	0.09	0.71	0.477

Note: MAP = mastery-approach goals; PAP = performance-approach goals; PAV = performance-avoidance goals; M-STR = mastery goal structures. * p < 0.05 ** *p ≤* 0.001.

**Table 7:** Descriptives of Surface and Adaptive Strategies based on final models.

Group	Mean	SE	df
Surface Strategies			
ND	3.76	0.024	529
LD	4.23	0.059	
RCD	3.98	0.097	
Adaptive Strategies			
ND	3.45	0.023	526
LD	2.95	0.059	
RCD	3.32	0.099	

## Data Availability

Not applicable.
